# MRI for nodal restaging after neoadjuvant therapy in rectal cancer with histopathologic comparison

**DOI:** 10.1186/s40644-023-00589-0

**Published:** 2023-07-13

**Authors:** Zhiwen Zhang, Yan Chen, Ziqiang Wen, Xuehan Wu, Yutao Que, Yuru Ma, Yunzhu Wu, Quanmeng Liu, Wenjie Fan, Shenping Yu

**Affiliations:** 1 Department of Radiology, The First Affiliated Hospital, Sun Yat-sen University, 510080 Guangzhou, China; 2 Department of Radiology, The Seventh Affiliated Hospital, Sun Yat-sen University, 518036 Shenzhen, China; 3 MR Scientific Marketing, SIEMENS Healthineers Ltd, 200124 Shanghai, China

**Keywords:** Rectal cancer, Neoadjuvant therapy, Lymph node, Magnetic resonance imaging, Histopathology

## Abstract

**Background:**

After neoadjuvant therapy, most of the lymph nodes (LNs) will shrink and disappear in patients with rectal cancer. However, LNs that are still detectable on MRI carry a risk of metastasis. This study aimed to evaluate the performance of the European Society of Gastrointestinal and Abdominal Radiology (ESGAR) criterion (short-axis diameter ≥ 5 mm) in diagnosing malignant LNs in patients with rectal cancer after neoadjuvant therapy, and whether nodal morphological characteristics (including shape, border, signal homogeneity, and enhancement homogeneity) could improve the diagnostic efficiency for LNs ≥ 5 mm.

**Methods:**

This retrospective study included 90 patients with locally advanced rectal cancer who underwent surgery after neoadjuvant therapy and performed preoperative MRI. Two radiologists independently measured the short-axis diameter of LNs and evaluated the morphological characteristics of LNs ≥ 5 mm in consensus. With a per node comparison with histopathology as the reference standard, a ROC curve was performed to evaluate the diagnostic performance of the size criterion. For categorical variables, either a *χ*^2^ test or Fisher’s exact test was used.

**Results:**

A total of 298 LNs were evaluated. The AUC for nodal size in determining nodal status was 0.81. With a size cutoff value of 5 mm, the sensitivity, specificity, positive predictive value, negative predictive value and accuracy were 65.9%, 87.0%, 46.8%, 93.6% and 83.9%, respectively. No significant differences were observed in any of the morphological characteristics between benign and malignant LNs ≥ 5 mm (all *P* > 0.05).

**Conclusions:**

The ESGAR criterion demonstrated moderate diagnostic performance in identifying malignant LNs in patients with rectal cancer after neoadjuvant therapy. It was effective in determining the status of LNs < 5 mm but not for LNs ≥ 5 mm, and the diagnostic efficiency could not be improved by considering nodal morphological characteristics.

## Background

Patients with locally advanced rectal cancer (LARC) and those who are unable to undergo radical resection are recommend to receive neoadjuvant therapy, which aims to increase the possibility of complete resection and reduce the risk of local recurrence by downstaging and downsizing [1]. After neoadjuvant therapy, most of the lymph nodes (LNs) shrink and disappear, but the LNs that can still be detected on MRI carry a risk of metastasis, with LNs larger than 5 mm having a 38.6% possibility of being pathologically metastatic [2]. Even in patients with a pathological complete response, 3.2-14% of visible LNs are malignant [3, 4]. Malignant LNs can lead to poor prognosis, including local recurrence and reduced long-term survival rate [1, 5]. Therefore, for patients after neoadjuvant therapy, particularly whose who are nearly clinical complete responders, MRI restaging of LNs is valuable in evaluating the feasibility of conservative treatment, such as a local excision or a watch-and-wait strategy.

Restaging of benign and malignant LNs is more challenging than primary staging in rectal cancer. Most studies recognize that MRI criteria for LNs involvement in primary staging include size, shape, border, signal homogeneity, and enhancement homogeneity [6–8]. However, these criteria are not entirely suitable for nodal restating after neoadjuvant therapy [9, 10]. Most research has primarily relied on the size criterion and has not considered whether morphological characteristics (including shape, border, signal homogeneity and enhancement homogeneity) can effectively distinguish benign and malignant LNs [4, 9, 11]. Only a few studies have shown that nodal morphological characteristics contribute to nodal restaging [12–14]. However, evaluating the details of morphological characteristics in small LNs (short-axis diameter < 5 mm) have been considered challenging [10].

In 2016, a new criterion was added to European Society of Gastrointestinal and Abdominal Radiology (ESGAR), considering LNs with a short-axis diameter < 5 mm as benign after neoadjuvant therapy [6]. To the best of our knowledge, no study has validated this criterion for a node-by-node evaluation. Thus, this study aimed to validate the ESGAR criterion and explore the diagnostic efficiency of nodal morphological characteristics for LNs ≥ 5 mm.

## Methods

### Patients

**Fig. 1:**
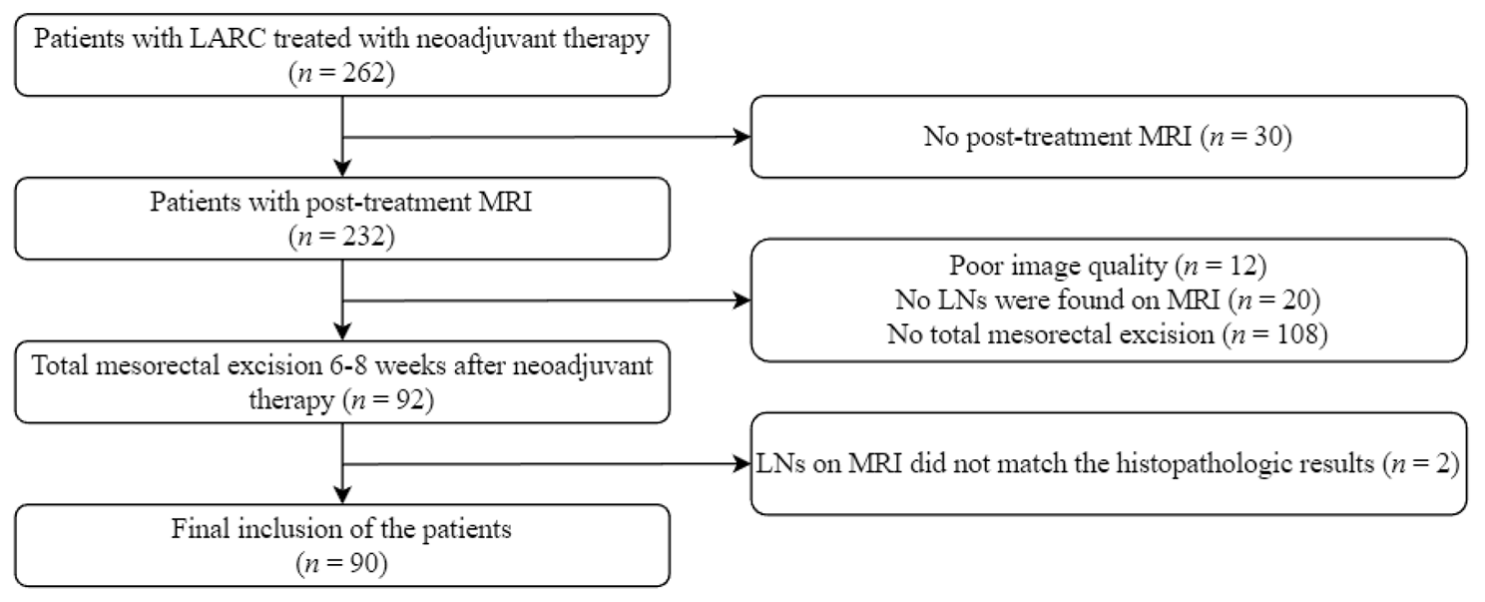
Flow diagram of patients included

This retrospective study was approved by our Institutional Review Board, and written informed consent was obtained from all patients. A total of 262 consecutive patients with LARC treated with neoadjuvant therapy between March 2015 and June 2021 were considered for inclusion. Among these patients, 172 patients were excluded for the following reasons (a) no total mesorectal excision 6–8 weeks after neoadjuvant therapy (*n* = 108); (b) no post-treatment MRI within 2 weeks before surgery (*n* = 30); (c) poor image quality for evaluation (*n* = 12); (d) no LNs found on MRI (*n* = 20); and (e) the location of the LNs on MRI did not match the histopathologic results (*n* = 2). Finally, 90 patients (68 men, 22 women; median age: 55 years, range: 20–90 years) were included in this study (Fig. 1).

### Neoadjuvant therapy

Neoadjuvant therapy included preoperative chemoradiotherapy and preoperative chemotherapy. Preoperative chemoradiotherapy regimen involved long-term radiotherapy using volumetric modulated arc therapy with a total dose of 50–60 Gy for primary and nodal gross tumor volumes administered with conventional segmentation, along with concurrent chemotherapy. Chemotherapy regimens included the mFOLFOX regimen (leucovorin + fluorouracil + oxaliplatin), CapeOX regimen (capecitabine + oxaliplatin), or capecitabine monotherapy.

### MRI examination

Patients, except those with lower and large rectal tumors, were infused with an appropriate amount (20–80 mL) of ultrasonic gel into the rectum. To reduce bowel motility, 20 mg of raceanisodamine hydrochloride was injected intramuscularly 10 min before MRI examination unless contraindicated.

MRI was performed using a 3.0-T scanner (Magnetom Verio, Siemens Healthcare) with a 6-channel phased-array body coil. Patients were positioned supine with feet first. The rectal MRI protocols included (a) sagittal, coronal and oblique axial (orthogonal to tumor base) non-fat-suppressed high-spatial-resolution T2- weighted imaging (HR-T2WI) using a turbo spin-echo sequence; and (b) coronal fat-suppressed isotropic contrast-enhanced three-dimensional high-spatial-resolution T1-weighted imaging (CE-3D-HR-T1WI) using a gradient-echo sequence. Axial, sagittal and coronal multiplanar reconstructions were performed on CE-3D-HR-T1WI with a slice thickness of 3 mm. An intravenous bolus of 0.2 mL/kg gadopentetate dimeglumine was injected at a rate of 3.0 mL/s, followed by a 25-mL saline flush at the same rate. Detailed protocols are listed in Table 1.

**Table 1:** High-spatial-resolution MRI protocols for rectal cancer

Sequences	TR/TE (ms)	Slice thickness/Gap (mm)	Slices	Base resolution	Phase resolution (%)	FOV (mm)	Voxel size (mm^3^)	Acquisition time
High-spatial-resolution T2-weighted imaging								
Sagittal	3000/87	3/0	19	320	80	180	0.7 × 0.6 × 3.0	2 min 30 s
Coronal	4000/77	3/0	25	384	80	220	0.7 × 0.6 × 3.0	2 min 52 s
Oblique axial	3000/84	3/0	24	320	100	180	0.6 × 0.6 × 3.0	3 min 18 s
Contrast-enhanced three-dimensional high-spatial-resolution T1-weighted imaging								
Coronal	10/4.9	1/0.2	144	384	100	380	1.0 × 1.0 × 1.0	3 min 10 s

*TR*, repetition time; *TE*, echo time; *FOV*, field of view

### Image interpretation

Two radiologists with 2 and 7 years of experience in rectal MRI, reviewed all MR images blinded to histopathologic findings. Firstly, all visible LNs on HR-T2WI were determined by the two radiologists in consensus. Secondly, the short-axis diameters of LNs were measured independently, and the average values were calculated for subsequent analyses. Thirdly, the two radiologists assessed the shape (oval/round), border (smooth/irregular), and signal homogeneity (homogeneous/heterogeneous) of LNs ≥ 5 mm on HR-T2WI and enhancement homogeneity (homogeneous/ heterogeneous) on CE-3D-HR-T1WI in consensus.

### Radiologic–histopathologic comparison

All visible regional LNs on HR-T2WI were divided into three groups, including mesorectal, superior rectal and inferior mesenteric LNs. Based on the agreed-upon nodal position by the radiologist and surgeon, an experienced surgeon specializing in colorectal cancer successively localized and removed the regional LNs in different groups during surgery. In order to provide an accurate node-by-node comparison between MR images and histopathological findings, special attention was paid to nodal size and morphology, as well as nodal relative position to the tumor, rectal wall, mesorectal fascia, vessels and adjacent LNs [15]. The excised LNs were then sent to the pathology department and quickly placed in individual trays. All LNs were fixed in formalin and stained with hematoxylin and eosin. Thereafter, an experienced gastrointestinal pathologist analyzed and classified each LN as benign or malignant under light microscope. The LNs reported by histopathology were rematched with HR-T2WI in the corresponding groups and were excluded if they could not be matched.

### Statistical analysis

Statistical analyses were performed using SPSS software (version 25.0, IBM). Figures were generated using GraphPad Prism (version 9.0, GraphPad Software) and MedCalc statistical software (version 15.8, MedCalc Software bvba, Ostend, Belgium). The normality of quantitative data was test by using Kolmogorov-Smirnov test. Normally distributed data were compared with the independent samples *t-*test, while nonnormally distributed data were presented as medians with ranges and compared with the Mann-Whitney *U* test. Categorical data were expressed as numbers with percentages and compared with the *χ*^2^ test or Fisher’s exact test. ROC curve was constructed, and the AUC with a 95% confidence interval was calculated to evaluate the diagnostic efficacy of nodal size. With a short-axis diameter cutoff value of 5 mm, the sensitivity, specificity, positive predictive value (PPV), negative predictive value (NPV) and accuracy were calculated. The morphological characteristics were compared for LNs ≥ 5 mm using the *χ*^2^ test or Fisher’s exact test. A two-tailed *P* value < 0.05 indicated a statistically significant difference.

## Results

### Clinicopathologic findings

A total of 90 patients were included, of which 27 (30.0%, 27/90) were confirmed to have malignant LNs. Malignant LNs were more likely occur in patients with pT3-4 tumors (*P* < 0.05) (Table 2). Histopathology of 1049 LNs harvested from the rectal specimens in 90 patients (median:11, range: 0–31) revealed that 72 (6.9%, 72/1049) were malignant. A total of 308 LNs were visualized on MRI in the 90 patients (median:3, range:1–8). For a node-by-node evaluation, 298 LNs were successfully matched between MRI and histopathology, of which 44 were malignant.

**Table 2:** Relationship between clinicopathologic features and lymph nodes metastases in 90 patients

Parameters	Total (*n* = 90)	ypN0 (*n* = 63)	ypN+ (*n* = 27)	*P* value
Age (years)	55 (24–82)^*^	56(27–82) ^*^	55(24–75) ^*^	0.926 ^a^
Sex				0.830 ^b^
Man	68 (75.6%)	48(76.2%)	20(74.1%)	
Woman	22 (24.4%)	15(23.8%)	7(25.9%)	
Tumor location^#^				0.777 ^c^
Upper	11(12.2%)	7(11.1%)	4(14.8%)	
Middle	51(56.7%)	35(55.6%)	16(59.3%)	
Lower	28(31.1%)	21(33.3%)	7(25.9%)	
Histological subtype				0.234 ^c^
Nonmucinous adenocarcinoma	82(91.1%)	59(93.7%)	23(85.2%)	
Mucinous adenocarcinoma	8(8.9%)	4(6.3%)	4(14.8.%)	
Differentiation				0.808 ^c^
Well	1(1.1%)	1(1.6%)	0(0.0%)	
Moderate	78(86.7%)	55(87.3%)	23(85.2%)	
Poor	11(12.2%)	7(11.1%)	4(14.8%)	
ypT stage				0.004 ^b^
ypT0-2	44(48.9%)	37(58.7%)	7(25.9%)	
ypT3-4	46(51.1%)	26(41.3%)	20(74.1%)	

^*^ Date are medians and ranges in parentheses

^#^ According to the distance from the most caudal border of the rectal tumor to the anal verge on MRI: upper, > 10 cm; middle, 5–10 cm; lower, < 5 cm

^a^ Mann-Whitney *U* test, ^b^*χ*^2^ test, ^c^ Fisher’s exact test

*yp*, post-neoadjuvant treatment pathological feature

### Diagnostic value of nodal size

**Fig. 2:**
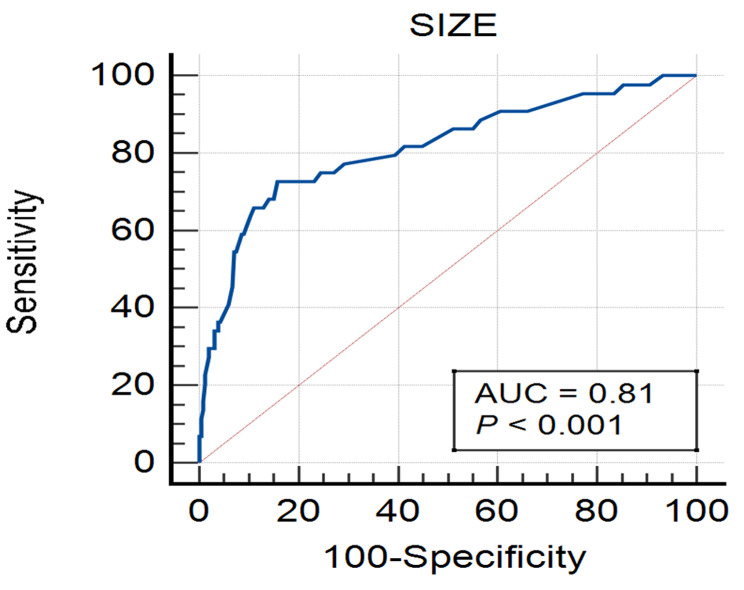
ROC curve showing the diagnostic performance of nodal size for malignant lymph nodes

**Fig. 3:**
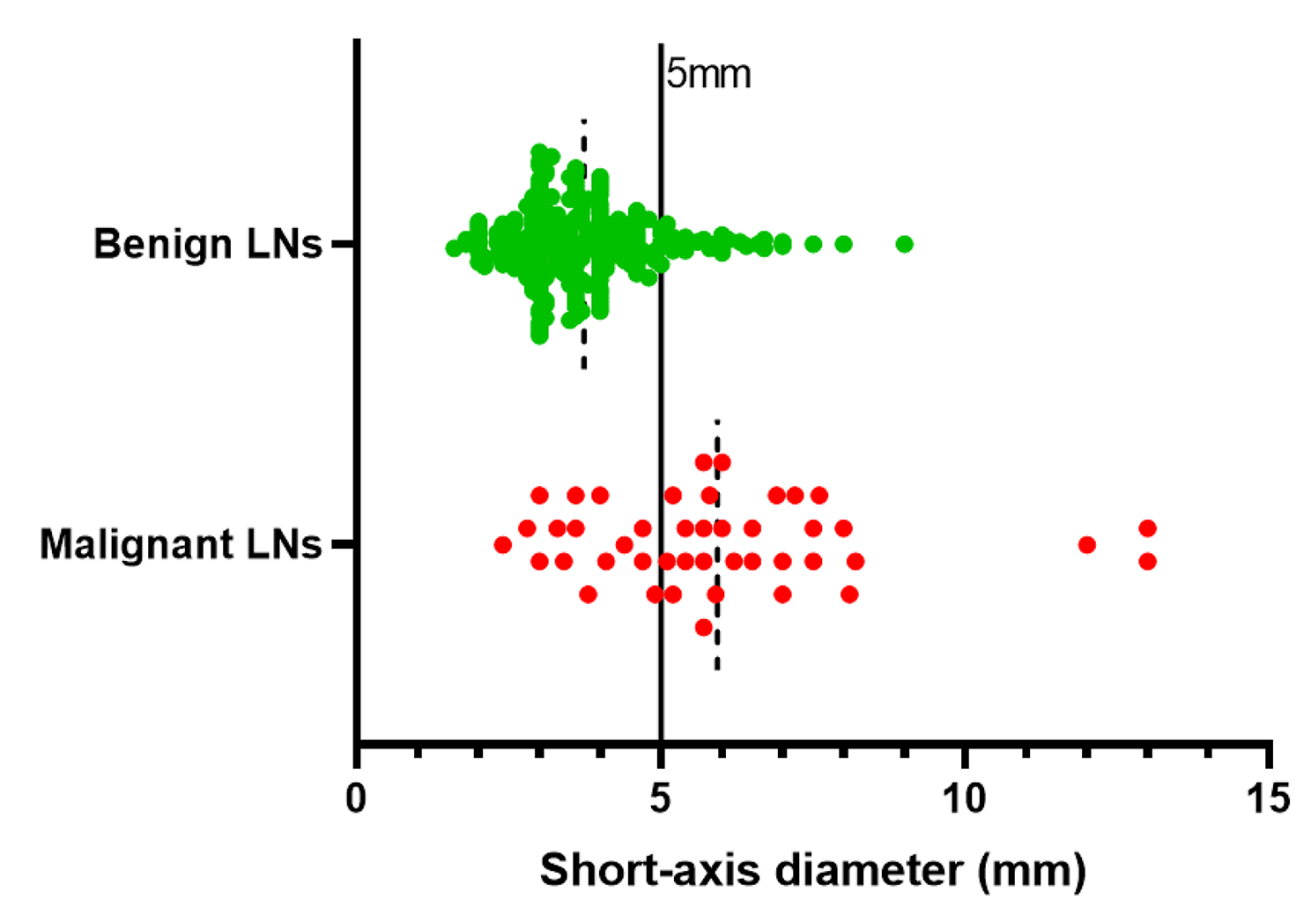
Short-axis diameter distribution of benign and malignant LNs. Dotted line: mean, solid line: cutoff value. 93.6% of the LNs < 5 mm were benign (green spots), and only 46.8% of the LNs ≥ 5 mm were malignant (red spots). *LNs*, lymph nodes

The median short-axis diameter was 3.7 mm (range: 1.6-9 mm) for the 254 benign LNs and 5.9 mm (range:2.4–13 mm) for the 44 malignant LNs. The AUC was 0.81 (95% confidence interval: 0.74–0.89), indicating that nodal size had moderate diagnostic performance in distinguishing malignant from benign LNs (Fig. 2). When LNs ≥ 5 mm were considered malignant, sensitivity, specificity, PPV, NPV and accuracy for determining malignant LNs were 65.9% (29/44), 87.0% (221/254), 46.8% (29/62), 93.6% (221/236) and 83.9% (250/298), respectively (Table 3). Among LNs < 5 mm, almost all (93.6%, 221/236) were benign, while less than half (46.8%, 29/62) LNs ≥ 5 mm were malignant (Fig. 3).

**Table 3:** Comparison of nodal size and histopathologic findings in 298 lymph nodes

	Histopathologic findings		
Nodal size	ypLN-	ypLN+	Total
LN- (< 5 mm)	221	15	236
LN+ (≥ 5 mm)	33	29	62
Total	254	44	298

*LNs*, lymph nodes; *yp*, post-neoadjuvant treatment pathological feature

### Nodal status compared with its morphological characteristics

**Fig. 4:**
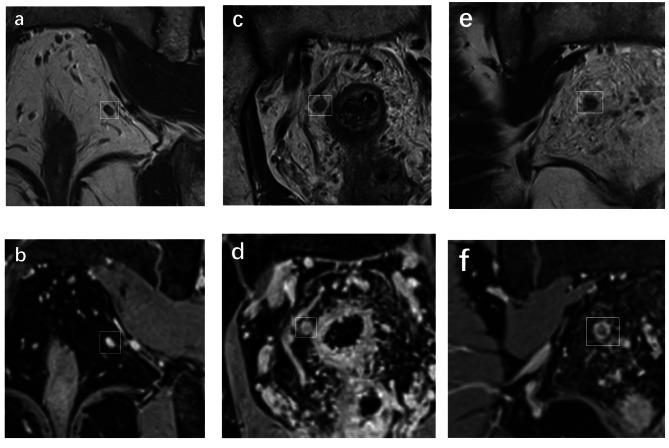
Coronal non-fat-suppressed high-spatial-resolution T2-weighted imaging (a, c and e); coronal fat-suppressed isotropic contrast-enhanced three-dimensional high-spatial-resolution T1-weighted imaging (b, d and f). The lymph nodes were signed by white boxes. **a-b**, Benign node with 3.7 mm in short-axis diameter, showed oval, smooth border, homogeneous signal and homogeneous enhancement. **c-d**, Benign node with 5.6 mm in short-axis diameter, showed round, irregular border, heterogeneous signal and heterogeneous enhancement. **e-f**, Malignant node with 5.8 mm in short-axis diameter, showed round, irregular border, heterogeneous signal and heterogeneous enhancement

Among the 62 LNs ≥ 5 mm, 21.2% (7/33) of benign LNs showed an oval shape, while 86.2% (25/29) of malignant LNs were round. Smooth borders were observed in 69.7% (23/33) of benign LNs, compared to only 17.2% (5/29) of malignant LNs with irregular borders. Homogeneous signal intensity was present in 66.7% (22/33) of benign LNs, while 41.4% (12/29) of malignant LNs showed heterogeneous signal intensity. Furthermore, 21.2% (7/33) of benign LNs exhibited homogeneous enhancement, compared to 86.2% (25/29) of malignant LNs with heterogeneous enhancement (Fig. 4). However, none of these morphological characteristics were found to be statistically significant in differentiating benign and malignant LNs (*P* > 0.05) (Table 4).

**Table 4:** Comparison of nodal morphological characteristics and histopathologic findings in 62 lymph nodes ≥ 5 mm

	Histopathologic findings		
Morphological characteristics	ypLN- (*n* = 33)	ypLN+ (*n* = 29)	*P* value
Shape			0.445 ^a^
Oval	7 (21.2%)	4 (13.8%)	
Round	26 (78.8%)	25 (86.2%)	
Border			0.231 ^a^
Smooth	23 (69.7%)	24 (82.8%)	
Irregular	10 (30.3%)	5 (17.2%)	
Signal homogeneity			0.513 ^a^
Homogeneous	22 (66.7%)	17 (58.6%)	
Heterogeneous	11 (33.3%)	12 (41.4%)	
Enhancement homogeneity			0.445 ^a^
Homogeneous	7 (21.2%)	4 (13.8%)	
Heterogeneous	26 (78.8%)	25 (86.2%)	

*LNs*, lymph nodes; *yp*, post-neoadjuvant treatment pathological feature

^a^*χ*^2^ test

## Discussion

For the evaluation of LNs in rectal cancer, many studies have focused on assessing the agreement between MRI and histopathology for N-staging [16, 17]. Although this method is easy to implement, it ignores the characteristics of each individual node. In our study, we conducted a node-by-node analysis, matching and verifying each LN found on MRI with the pathological results. This approach has been proven to be more specific and valuable in establishing diagnostic criteria for LNs [7, 18].

Our study validated that the ESGAR criterion had a moderate diagnostic performance (AUC = 0.81) for nodal restaging in rectal cancer after neoadjuvant therapy. With a short-axis diameter cutoff value of 5 mm, the sensitivity, specificity and accuracy were 65.9%, 87.0% and 83.9%, respectively. The size criterion is the most widely used indicator for nodal staging and restaging in current studies on rectal cancer LNs [9, 19]. According to the ESGAR consensus meeting in 2012, the size criterion after neoadjuvant therapy is more reliable than the baseline MRI assessment for diagnosing malignant LNs [19]. Many studies have shown that the number of LNs usually decreases, and the short-axis diameter becomes smaller or even disappears after neoadjuvant therapy. Downsized LNs have a very low chance of metastasis [2]. However, there is still no consensus on the optimal cutoff value. In the 2016 ESGAR, a new item was added for MRI nodal restaging: all LNs < 5 mm should be considered benign, but there are no reliable criteria for LNs ≥ 5 mm. Our study also showed that short-axis diameter cutoff value of 5 mm had a high NPV, as almost all (93.6%, 221/236) LNs < 5 mm were benign. Thus, we can confidently conclude that there are no malignant LNs if all LNs on MRI after neoadjuvant therapy are < 5 mm. However, among the 62 LNs ≥ 5 mm, malignant LNs accounted for only 46.8% (29/62), resulting a low PPV. Therefore, it is impossible to effectively distinguish benign and malignant LNs ≥ 5 mm.

Although we used HR-T2WI and CE-3D-HR-T1WI with thin thickness in our study, confidently observing the morphological features of small LNs remains challenging [9, 10]. Consequently, we only evaluated the morphological features of LNs ≥ 5 mm. Although morphological characteristics were effective in predicting the status of LNs before treatment [18], we found no significant differences in shape, border, signal homogeneity, and enhancement homogeneity between benign and malignant LNs after neoadjuvant therapy. Nodal shape is likely influenced by the scanning planes. Even benign LNs showed irregular border and heterogeneous signal intensity. This could be attributed to fibrous thickening of the capsule and fibrotic or mucinous changes after treatment, which make elevation more complex [20]. Regarding enhancement homogeneity evaluation, we applied fat-suppressed isotropic CE-3D-HR-T1WI, which has demonstrated good performance in nodal staging [7]. Compared with HR-T2WI, this sequence offers advantages such as higher spatial resolution, improved signal-to-noise ratio, and multiplanar reconstruction, providing better-detailed of LNs. However, heterogenous enhancement could still be observed in benign LNs ≥ 5 mm, likely due to nodal fibrosis or the presence of acellular mucin lakes caused by neoadjuvant therapy [20, 21]. Malignant LNs with smooth border, homogeneous signal intensity, and enhancement may contain micrometastases.

There were some limitations in our study. Firstly, we only evaluated the morphological features of LNs ≥ 5 mm because accurately assessing the morphological features of small LNs is challenging. Secondly, the number of metastatic LNs was lower than that of benign because patients with obvious LN metastasis usually do not undergo surgery. Finally, we did not evaluate iliac LNs as extended pelvic lymphadenectomy is not usually performed in total mesorectal excision.

## Conclusions

In summary, our study demonstrates that the ESGAR has moderate diagnostic performance for nodal restaging in rectal cancer after neoadjuvant therapy. LNs < 5 mm can be effectively identified as benign using the size criterion alone, in line with the ESGAR recommendation. However, morphological features do not aid in the diagnosis of LNs ≥ 5 mm on MRI restaging. Future research should focus on refining the criteria for distinguishing benign and malignant LNs ≥ 5 mm and exploring alternative imaging techniques to improve the accuracy of nodal restaging in rectal cancer after neoadjuvant therapy.

## Data Availability

The datasets used and/or analysed during the current study are available from the corresponding author on reasonable request.

## List of abbreviations

LARC: locally advanced rectal cancer
LNs: lymph nodes
ESGAR: European Society of Gastrointestinal and Abdominal Radiology
HR-T2WI: high-spatial-resolution T2-weighted imaging
CE-3D-HR-T1WI: contrast-enhanced three-dimensional high-spatial-resolution T1-weighted imaging
PPV: positive predictive value
NPV: negative predictive value
